# Withdrawal: A phosphate transporter from the root endophytic fungus *Piriformospora indica* plays a role in phosphate transport to the host plant

**DOI:** 10.1016/j.jbc.2021.100457

**Published:** 2021-03-27

**Authors:** Vikas Yadav, Manoj Kumar, Deepak Kumar Deep, Hemant Kumar, Ruby Sharma, Takshashila Tripathi, Narendra Tuteja, Ajay Kumar Saxena, Atul Kumar Johri

VOLUME 285 (2010) PAGES 26532–26544

This article has been withdrawn by the authors except Ajay Kumar Saxena and Deepak Kumar Deep who could not be contacted. Fig. 5*C* panel 3 is duplicated in Fig. 10*A*, b iii. Fig. 10*A*, a iii is duplicated as Fig. 1 in *Microbiology* (2009), 155: 780–790. Fig. 5*C* panel 6 is duplicated as Fig. 1*A*, b ii right, Fig. 10*A*, a iii is duplicated as Fig. 1*A*, a ii right, and Fig. 10*A*, c is duplicated in Fig. 1*A*, d in *Plant Signaling & Behavior* (2011) 6(5): 723–725, respectively representing different experimental conditions. *Plant Signaling & Behavior* as well as *Microbiology* have replaced and corrected the figures which were overlapping with the *JBC* figures as mentioned above. Both *Plant Signaling & Behavior* and *Microbiology* have published a corrigendum related to the respective figures.

